# Crystal structure of tetra­aqua­bis­(thio­cyanato-κ*N*)nickel(II)–2,5-di­methyl­pyrazine (1/4)

**DOI:** 10.1107/S2056989014026991

**Published:** 2015-01-03

**Authors:** Stefan Suckert, Mario Wriedt, Inke Jess, Christian Näther

**Affiliations:** a Institut für Anorganische Chemie, Christian-Albrechts-Universität Kiel, Max-Eyth-Strasse 2, 24118 Kiel, Germany; b Department of Chemistry & Biomolecular Science, Clarkson University, Potsdam, NY 13699, USA

**Keywords:** crystal structure, thio­cyanat, nickel(II) complex, 2,5-di­methyl­pyrazine, hydrogen bonding

## Abstract

In the crystal structure of the title compound, [Ni(NCS)_2_(H_2_O)_4_]·4C_6_H_8_N_2_, the Ni^II^ cations are coordinated by four water ligands and two *trans*-coordinated terminally *N*-bonded thio­cyanate anions in a slightly distorted octa­hedral geometry. The asymmetric unit consists of a Ni^2+^ cation located on a centre of inversion, two water mol­ecules and one thio­cyanate ligand, as well as two uncoordinated 2,5-di­methyl­pyrazine ligands in general positions. In the crystal, discrete complex mol­ecules are linked into a three-dimensional network by O—H⋯N hydrogen bonding between the water H atoms and the 2,5-di­methyl­pyrazine N atoms.

## Related literature   

For background information on the design and preparation of coordination polymers, see Näther *et al.* (2013 ▸). For a different structure with thio­cyanates and 2,5-di­methyl­pyrazine, see: Otieno *et al.* (2003 ▸).
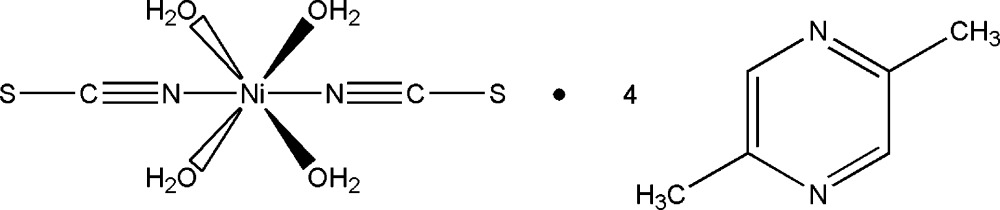


## Experimental   

### Crystal data  

[Ni(NCS)_2_(H_2_O)_4_]·4C_6_H_8_N_2_
*M* *_r_* = 679.51
Orthorhombic, 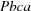

*a* = 13.0731 (6) Å
*b* = 14.7989 (8) Å
*c* = 17.3092 (11) Å
*V* = 3348.8 (3) Å^3^
*Z* = 4
Mo *K*α radiation
μ = 0.75 mm^−1^
*T* = 170 K
0.12 × 0.10 × 0.08 mm

### Data collection  

Stoe IPDS-1 diffractometer
Absorption correction: numerical (*X-SHAPE* and *X-RED32*; Stoe & Cie, 2008 ▸) *T*_min_ = 0.912, *T*_max_ = 0.938
21266 measured reflections
4041 independent reflections
3146 reflections with *I* > 2σ(*I*)
*R*_int_ = 0.035

### Refinement  

*R*[*F* ^2^ > 2σ(*F* ^2^)] = 0.036
*wR*(*F* ^2^) = 0.100
*S* = 1.02
4041 reflections
201 parameters
H-atom parameters constrained
Δρ_max_ = 0.35 e Å^−3^
Δρ_min_ = −0.40 e Å^−3^

### 

Data collection: *X-AREA* (Stoe & Cie, 2008 ▸); cell refinement: *X-AREA*; data reduction: *X-AREA*; program(s) used to solve structure: *SHELXS97* (Sheldrick, 2008 ▸); program(s) used to refine structure: *SHELXL97* (Sheldrick, 2008 ▸); molecular graphics: *XP* in *SHELXTL* (Sheldrick, 2008 ▸) and *DIAMOND* (Brandenburg, 1999 ▸); software used to prepare material for publication: *publCIF* (Westrip, 2010 ▸).

## Supplementary Material

Crystal structure: contains datablock(s) I, global. DOI: 10.1107/S2056989014026991/pk2540sup1.cif

Structure factors: contains datablock(s) I. DOI: 10.1107/S2056989014026991/pk2540Isup2.hkl

**Fig. 1.**
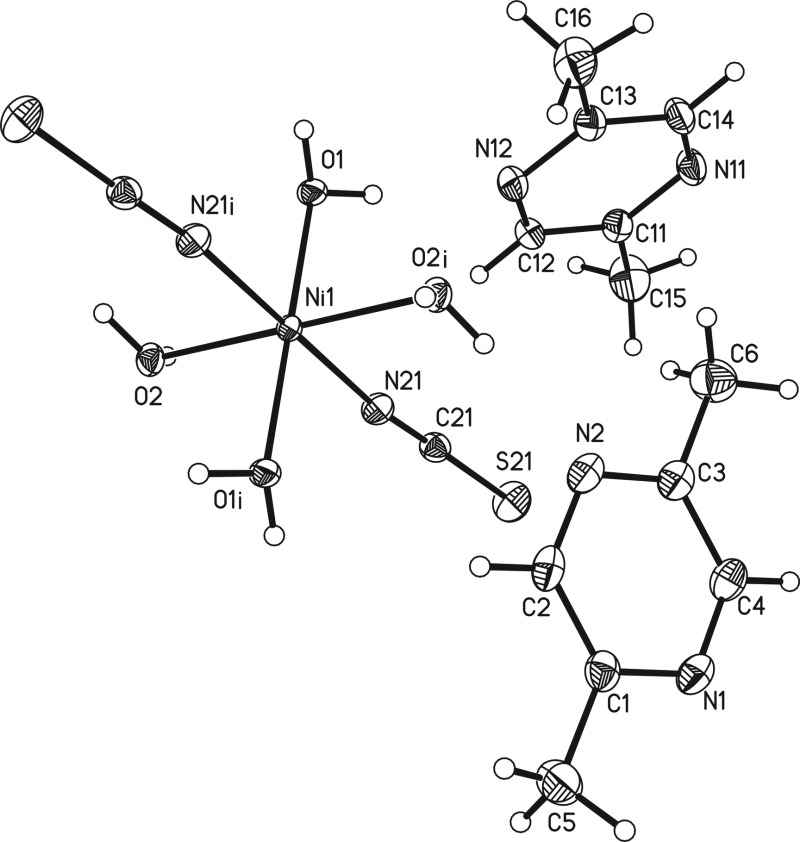
Part of the crystal structure of the title compound with labelling and displacement ellipsoids drawn at the 50% probability level. Symmetry code: i = x+1,-y+1,-z+1.

**Fig. 2.**
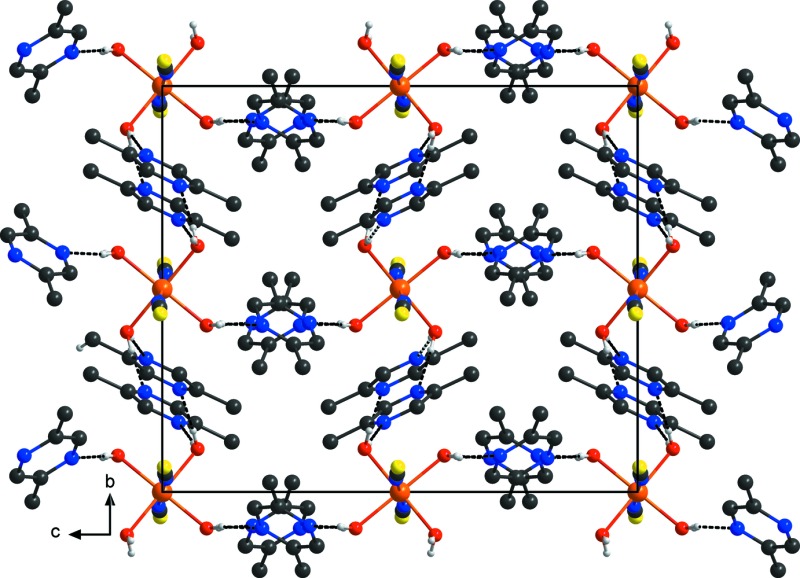
Crystal structure of the title compound with view along the crystallographic a axis. Hydrogen bonding is shown as dashed lines and for clarity only the O-H H atoms are shown.

. DOI: 10.1107/S2056989014026991/pk2540fig1.tif
Part of the crystal structure of the title compound with labelling and displacement ellipsoids drawn at the 50% probability level. Symmetry code: i = x+1,-y+1,-z+1.

a . DOI: 10.1107/S2056989014026991/pk2540fig2.tif
Crystal structure of the title compound with view along the crystallographic *a* axis. Hydrogen bonding is shown as dashed lines and for clarity only the O-H H atoms are shown.

CCDC reference: 1038309

Additional supporting information:  crystallographic information; 3D view; checkCIF report

## Figures and Tables

**Table 1:** Hydrogen-bond geometry (, )

*D*H*A*	*D*H	H*A*	*D* *A*	*D*H*A*
O1H1*O*1N12	0.84	1.99	2.8284(18)	174
O1H2*O*1N11^i^	0.84	2.06	2.8963(18)	173
O2H1*O*2N2^ii^	0.84	2.00	2.8286(19)	169
O2H2*O*2N1^iii^	0.84	2.03	2.8665(19)	176

Symmetry codes: (i) 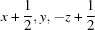; (ii) 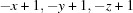; (iii) 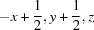.

## supplementary crystallographic information

### S1. Synthesis and crystallization

NiSO_4_.6 H_2_O and 2,5-di­methyl­pyrazine were purchased from Merck and Ba(NCS)_2_.3 H_2_O was purchased from Alfa Aesar. Ni(NCS)_2_ was synthesized by stirring 17.5 g (56.91 mmol) Ba(NCS)_2_.3 H_2_O and 15.00 g (57.03 mmol) NiSO_4_.6 H_2_O in 500 ml H_2_O at RT for two hours. The white residue of BaSO_4_ was filtered of and the solvent removed with a rotary evaporator. The homogeneity of the product was investigated by X-ray powder diffraction and elemental analysis. The title compound was prepared by the reaction of (0.15 mmol) 27.8 mg Ni(NCS)_2_ and (0.9 mmol) 97.5 µl 2,5-di­methyl­pyrazine at RT. After a few days blue block shaped crystals of the title compound were obtained.

### S2. Refinement

The C—H H atoms were positioned with idealized geometry (methyl H atoms allowed to rotate but not to tip) and were refined using a riding model with C—H = 0.95 Å for aromatic and C—H = 0.98 Å for methyl. Water hydrogen atoms were found in difference-electron density maps and fixed (SHELXL command AFIX 3). U_iso_(H) values were set to either 1.2U_eq_ or 1.5U_eq_ (-CH_3_, H_2_O) of the attached parent atom.

### Crystal data

**Table:**

[Ni(NCS)_2_(H_2_O)_4_]·4C_6_H_8_N_2_	*F*(000) = 1432
*M**_r_* = 679.51	*D*_x_ = 1.348 Mg m^−^^3^
Orthorhombic, *P**b**c**a*	Mo *K*α radiation, λ = 0.71073 Å
Hall symbol: -P 2ac 2ab	Cell parameters from 21266 reflections
*a* = 13.0731 (6) Å	θ = 2.8–28.1°
*b* = 14.7989 (8) Å	µ = 0.75 mm^−^^1^
*c* = 17.3092 (11) Å	*T* = 170 K
*V* = 3348.8 (3) Å^3^	Block, blue
*Z* = 4	0.12 × 0.10 × 0.08 mm

### Data collection

**Table:**

Stoe IPDS-1 diffractometer	4041 independent reflections
Radiation source: fine-focus sealed tube	3146 reflections with *I* > 2σ(*I*)
Graphite monochromator	*R*_int_ = 0.035
φ scans	θ_max_ = 28.1°, θ_min_ = 2.8°
Absorption correction: numerical (*X-SHAPE* and *X-RED32*; Stoe & Cie, 2008)	*h* = −17→15
*T*_min_ = 0.912, *T*_max_ = 0.938	*k* = −19→19
21266 measured reflections	*l* = −22→22

### Refinement

**Table:**

Refinement on *F*^2^	Secondary atom site location: difference Fourier map
Least-squares matrix: full	Hydrogen site location: inferred from neighbouring sites
*R*[*F*^2^ > 2σ(*F*^2^)] = 0.036	H-atom parameters constrained
*wR*(*F*^2^) = 0.100	*w* = 1/[σ^2^(*F*_o_^2^) + (0.0627*P*)^2^ + 0.7837*P*] where *P* = (*F*_o_^2^ + 2*F*_c_^2^)/3
*S* = 1.02	(Δ/σ)_max_ = 0.001
4041 reflections	Δρ_max_ = 0.35 e Å^−^^3^
201 parameters	Δρ_min_ = −0.40 e Å^−^^3^
0 restraints	Extinction correction: *SHELXL97* (Sheldrick, 2008), Fc*=kFc[1+0.001xFc^2^λ^3^/sin(2θ)]^-1/4^
Primary atom site location: structure-invariant direct methods	Extinction coefficient: 0.0068 (8)

### Special details

**Table:**

Geometry. All esds (except the esd in the dihedral angle between two l.s. planes) are estimated using the full covariance matrix. The cell esds are taken into account individually in the estimation of esds in distances, angles and torsion angles; correlations between esds in cell parameters are only used when they are defined by crystal symmetry. An approximate (isotropic) treatment of cell esds is used for estimating esds involving l.s. planes.
Refinement. Refinement of F^2^ against ALL reflections. The weighted R-factor wR and goodness of fit S are based on F^2^, conventional R-factors R are based on F, with F set to zero for negative F^2^. The threshold expression of F^2^ > 2sigma(F^2^) is used only for calculating R-factors(gt) etc. and is not relevant to the choice of reflections for refinement. R-factors based on F^2^ are statistically about twice as large as those based on F, and R- factors based on ALL data will be even larger.

### Fractional atomic coordinates and isotropic or equivalent isotropic displacement parameters (Å^2^)

**Table:**

	*x*	*y*	*z*	*U*_iso_*/*U*_eq_	
Ni1	0.5000	0.5000	0.5000	0.01215 (11)	
N21	0.34876 (11)	0.53353 (10)	0.50818 (8)	0.0178 (3)	
C21	0.26112 (13)	0.54542 (11)	0.50572 (8)	0.0161 (3)	
S21	0.13689 (3)	0.56120 (4)	0.50416 (3)	0.02775 (13)	
O1	0.51049 (8)	0.58972 (8)	0.40668 (6)	0.0160 (2)	
H1O1	0.4561	0.5882	0.3809	0.024*	
H2O1	0.5589	0.5854	0.3750	0.024*	
O2	0.54012 (9)	0.60597 (8)	0.57432 (6)	0.0162 (2)	
H1O2	0.6015	0.6200	0.5663	0.024*	
H2O2	0.5025	0.6507	0.5650	0.024*	
N1	0.09222 (12)	0.25396 (10)	0.53656 (9)	0.0231 (3)	
N2	0.26379 (12)	0.32843 (10)	0.46598 (9)	0.0223 (3)	
C1	0.18340 (14)	0.25266 (11)	0.57207 (10)	0.0211 (3)	
C2	0.26824 (14)	0.28976 (12)	0.53578 (11)	0.0227 (4)	
H2	0.3323	0.2876	0.5616	0.027*	
C3	0.17233 (14)	0.33146 (11)	0.43120 (10)	0.0210 (3)	
C4	0.08758 (14)	0.29358 (12)	0.46734 (11)	0.0235 (4)	
H4	0.0234	0.2960	0.4416	0.028*	
C5	0.18951 (17)	0.20962 (14)	0.65016 (11)	0.0321 (4)	
H5A	0.1634	0.2517	0.6892	0.048*	
H5B	0.2609	0.1947	0.6618	0.048*	
H5C	0.1483	0.1543	0.6506	0.048*	
C6	0.16595 (17)	0.37609 (14)	0.35372 (11)	0.0328 (4)	
H6A	0.1624	0.4418	0.3605	0.049*	
H6B	0.1045	0.3551	0.3267	0.049*	
H6C	0.2267	0.3606	0.3233	0.049*	
N11	0.16959 (11)	0.58969 (10)	0.20976 (8)	0.0228 (3)	
N12	0.33677 (12)	0.58344 (10)	0.30944 (8)	0.0211 (3)	
C11	0.16897 (13)	0.63643 (11)	0.27609 (10)	0.0208 (3)	
C12	0.25374 (14)	0.63271 (12)	0.32501 (9)	0.0203 (3)	
H12	0.2523	0.6668	0.3715	0.024*	
C13	0.33614 (14)	0.53519 (12)	0.24377 (10)	0.0207 (3)	
C14	0.25231 (14)	0.53952 (13)	0.19461 (10)	0.0225 (4)	
H14	0.2538	0.5053	0.1482	0.027*	
C15	0.07737 (16)	0.69264 (15)	0.29538 (13)	0.0374 (5)	
H15A	0.0305	0.6935	0.2512	0.056*	
H15B	0.0992	0.7545	0.3072	0.056*	
H15C	0.0424	0.6668	0.3403	0.056*	
C16	0.42685 (16)	0.47645 (15)	0.22664 (13)	0.0332 (4)	
H16A	0.4875	0.5144	0.2194	0.050*	
H16B	0.4140	0.4417	0.1795	0.050*	
H16C	0.4383	0.4349	0.2699	0.050*	

### Atomic displacement parameters (Å^2^)

**Table:**

	*U*^11^	*U*^22^	*U*^33^	*U*^12^	*U*^13^	*U*^23^
Ni1	0.00735 (16)	0.01786 (16)	0.01122 (15)	0.00033 (10)	−0.00024 (9)	0.00008 (10)
N21	0.0123 (7)	0.0226 (7)	0.0185 (7)	0.0012 (5)	0.0013 (5)	−0.0007 (5)
C21	0.0155 (8)	0.0178 (8)	0.0150 (7)	0.0015 (6)	0.0008 (6)	−0.0004 (6)
S21	0.0102 (2)	0.0405 (3)	0.0326 (3)	0.00601 (18)	−0.00014 (16)	−0.00175 (19)
O1	0.0108 (5)	0.0242 (6)	0.0131 (5)	−0.0002 (4)	0.0000 (4)	0.0033 (4)
O2	0.0121 (5)	0.0196 (5)	0.0170 (5)	0.0007 (4)	−0.0014 (4)	−0.0024 (4)
N1	0.0170 (7)	0.0221 (7)	0.0300 (8)	−0.0023 (6)	0.0024 (6)	−0.0015 (6)
N2	0.0178 (7)	0.0197 (7)	0.0293 (7)	−0.0027 (6)	0.0026 (6)	−0.0032 (6)
C1	0.0228 (8)	0.0178 (7)	0.0228 (8)	−0.0007 (7)	−0.0004 (6)	−0.0046 (6)
C2	0.0167 (8)	0.0225 (8)	0.0291 (9)	−0.0016 (7)	−0.0039 (7)	−0.0045 (7)
C3	0.0196 (8)	0.0171 (7)	0.0262 (8)	0.0017 (7)	0.0014 (7)	−0.0031 (6)
C4	0.0149 (8)	0.0251 (8)	0.0306 (9)	0.0005 (7)	−0.0018 (7)	0.0001 (7)
C5	0.0392 (12)	0.0327 (10)	0.0245 (9)	−0.0022 (9)	−0.0025 (8)	0.0009 (7)
C6	0.0359 (11)	0.0327 (10)	0.0298 (10)	0.0014 (9)	−0.0009 (8)	0.0057 (8)
N11	0.0184 (7)	0.0299 (8)	0.0201 (7)	0.0006 (6)	−0.0050 (6)	−0.0042 (6)
N12	0.0207 (7)	0.0256 (7)	0.0170 (6)	−0.0020 (6)	−0.0046 (5)	0.0009 (5)
C11	0.0189 (8)	0.0226 (8)	0.0209 (8)	−0.0012 (7)	−0.0006 (6)	−0.0015 (6)
C12	0.0226 (8)	0.0234 (8)	0.0151 (7)	−0.0032 (7)	−0.0009 (6)	−0.0023 (6)
C13	0.0184 (8)	0.0241 (8)	0.0198 (8)	−0.0004 (7)	−0.0023 (6)	−0.0006 (6)
C14	0.0211 (9)	0.0290 (9)	0.0173 (7)	0.0001 (7)	−0.0033 (7)	−0.0060 (6)
C15	0.0285 (11)	0.0413 (12)	0.0426 (12)	0.0099 (9)	−0.0016 (9)	−0.0125 (9)
C16	0.0228 (10)	0.0382 (10)	0.0386 (11)	0.0087 (9)	−0.0051 (8)	−0.0071 (9)

### Geometric parameters (Å, º)

**Table:**

Ni1—N21	2.0434 (15)	C5—H5A	0.9800
Ni1—N21^i^	2.0434 (15)	C5—H5B	0.9800
Ni1—O2	2.0951 (11)	C5—H5C	0.9800
Ni1—O2^i^	2.0951 (11)	C6—H6A	0.9800
Ni1—O1	2.0954 (11)	C6—H6B	0.9800
Ni1—O1^i^	2.0954 (11)	C6—H6C	0.9800
N21—C21	1.160 (2)	N11—C14	1.338 (2)
C21—S21	1.6411 (18)	N11—C11	1.340 (2)
O1—H1O1	0.8399	N12—C12	1.335 (2)
O1—H2O1	0.8400	N12—C13	1.342 (2)
O2—H1O2	0.8399	C11—C12	1.396 (2)
O2—H2O2	0.8400	C11—C15	1.496 (3)
N1—C4	1.335 (3)	C12—H12	0.9500
N1—C1	1.341 (2)	C13—C14	1.389 (2)
N2—C2	1.338 (2)	C13—C16	1.500 (3)
N2—C3	1.339 (2)	C14—H14	0.9500
C1—C2	1.388 (2)	C15—H15A	0.9800
C1—C5	1.496 (3)	C15—H15B	0.9800
C2—H2	0.9500	C15—H15C	0.9800
C3—C4	1.390 (2)	C16—H16A	0.9800
C3—C6	1.497 (3)	C16—H16B	0.9800
C4—H4	0.9500	C16—H16C	0.9800
			
N21—Ni1—N21^i^	180.0	C1—C5—H5B	109.5
N21—Ni1—O2	91.03 (5)	H5A—C5—H5B	109.5
N21^i^—Ni1—O2	88.97 (5)	C1—C5—H5C	109.5
N21—Ni1—O2^i^	88.97 (5)	H5A—C5—H5C	109.5
N21^i^—Ni1—O2^i^	91.03 (5)	H5B—C5—H5C	109.5
O2—Ni1—O2^i^	180.00 (4)	C3—C6—H6A	109.5
N21—Ni1—O1	87.87 (5)	C3—C6—H6B	109.5
N21^i^—Ni1—O1	92.13 (5)	H6A—C6—H6B	109.5
O2—Ni1—O1	89.01 (5)	C3—C6—H6C	109.5
O2^i^—Ni1—O1	90.99 (5)	H6A—C6—H6C	109.5
N21—Ni1—O1^i^	92.13 (5)	H6B—C6—H6C	109.5
N21^i^—Ni1—O1^i^	87.87 (5)	C14—N11—C11	117.34 (15)
O2—Ni1—O1^i^	90.99 (5)	C12—N12—C13	117.16 (15)
O2^i^—Ni1—O1^i^	89.01 (5)	N11—C11—C12	119.63 (16)
O1—Ni1—O1^i^	180.00 (5)	N11—C11—C15	118.88 (16)
C21—N21—Ni1	171.90 (14)	C12—C11—C15	121.48 (16)
N21—C21—S21	178.72 (15)	N12—C12—C11	123.00 (15)
Ni1—O1—H1O1	109.7	N12—C12—H12	118.5
Ni1—O1—H2O1	120.4	C11—C12—H12	118.5
H1O1—O1—H2O1	106.7	N12—C13—C14	119.93 (16)
Ni1—O2—H1O2	108.8	N12—C13—C16	118.09 (16)
Ni1—O2—H2O2	108.9	C14—C13—C16	121.97 (16)
H1O2—O2—H2O2	109.5	N11—C14—C13	122.91 (16)
C4—N1—C1	117.25 (15)	N11—C14—H14	118.5
C2—N2—C3	117.32 (15)	C13—C14—H14	118.5
N1—C1—C2	119.81 (16)	C11—C15—H15A	109.5
N1—C1—C5	117.87 (17)	C11—C15—H15B	109.5
C2—C1—C5	122.31 (17)	H15A—C15—H15B	109.5
N2—C2—C1	122.89 (16)	C11—C15—H15C	109.5
N2—C2—H2	118.6	H15A—C15—H15C	109.5
C1—C2—H2	118.6	H15B—C15—H15C	109.5
N2—C3—C4	119.71 (17)	C13—C16—H16A	109.5
N2—C3—C6	117.85 (17)	C13—C16—H16B	109.5
C4—C3—C6	122.44 (17)	H16A—C16—H16B	109.5
N1—C4—C3	122.99 (17)	C13—C16—H16C	109.5
N1—C4—H4	118.5	H16A—C16—H16C	109.5
C3—C4—H4	118.5	H16B—C16—H16C	109.5
C1—C5—H5A	109.5		

Symmetry code: (i) −*x*+1, −*y*+1, −*z*+1.

### Hydrogen-bond geometry (Å, º)

**Table:**

*D*—H···*A*	*D*—H	H···*A*	*D*···*A*	*D*—H···*A*
O1—H1*O*1···N12	0.84	1.99	2.8284 (18)	174
O1—H2*O*1···N11^ii^	0.84	2.06	2.8963 (18)	173
O2—H1*O*2···N2^i^	0.84	2.00	2.8286 (19)	169
O2—H2*O*2···N1^iii^	0.84	2.03	2.8665 (19)	176

Symmetry codes: (i) −*x*+1, −*y*+1, −*z*+1; (ii) *x*+1/2, *y*, −*z*+1/2; (iii) −*x*+1/2, *y*+1/2, *z*.
