# Supplementary figures and images for: Crystal structure of tetra­aqua­bis­(thio­cyanato-κN)nickel(II)–2,5-di­methyl­pyrazine (1/4)
Source: Acta Crystallogr E Crystallogr Commun. 2015 Jan 3;71(Pt 2):m18. doi: 10.1107/S2056989014026991 (PMC4384548; doi:10.1107/S2056989014026991)

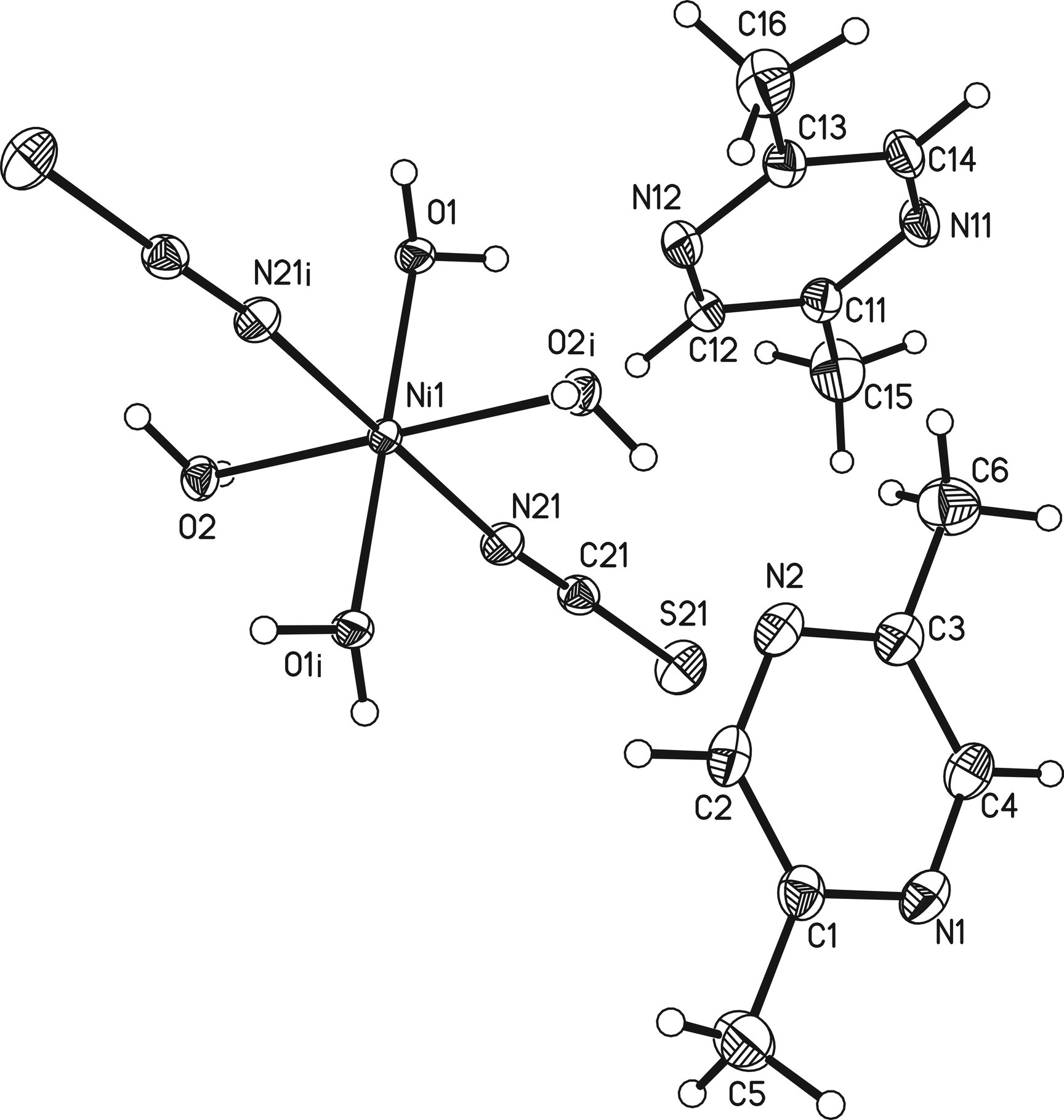

Supplement: Supplementary file 3 [file e-71-00m18-fig1.tif]

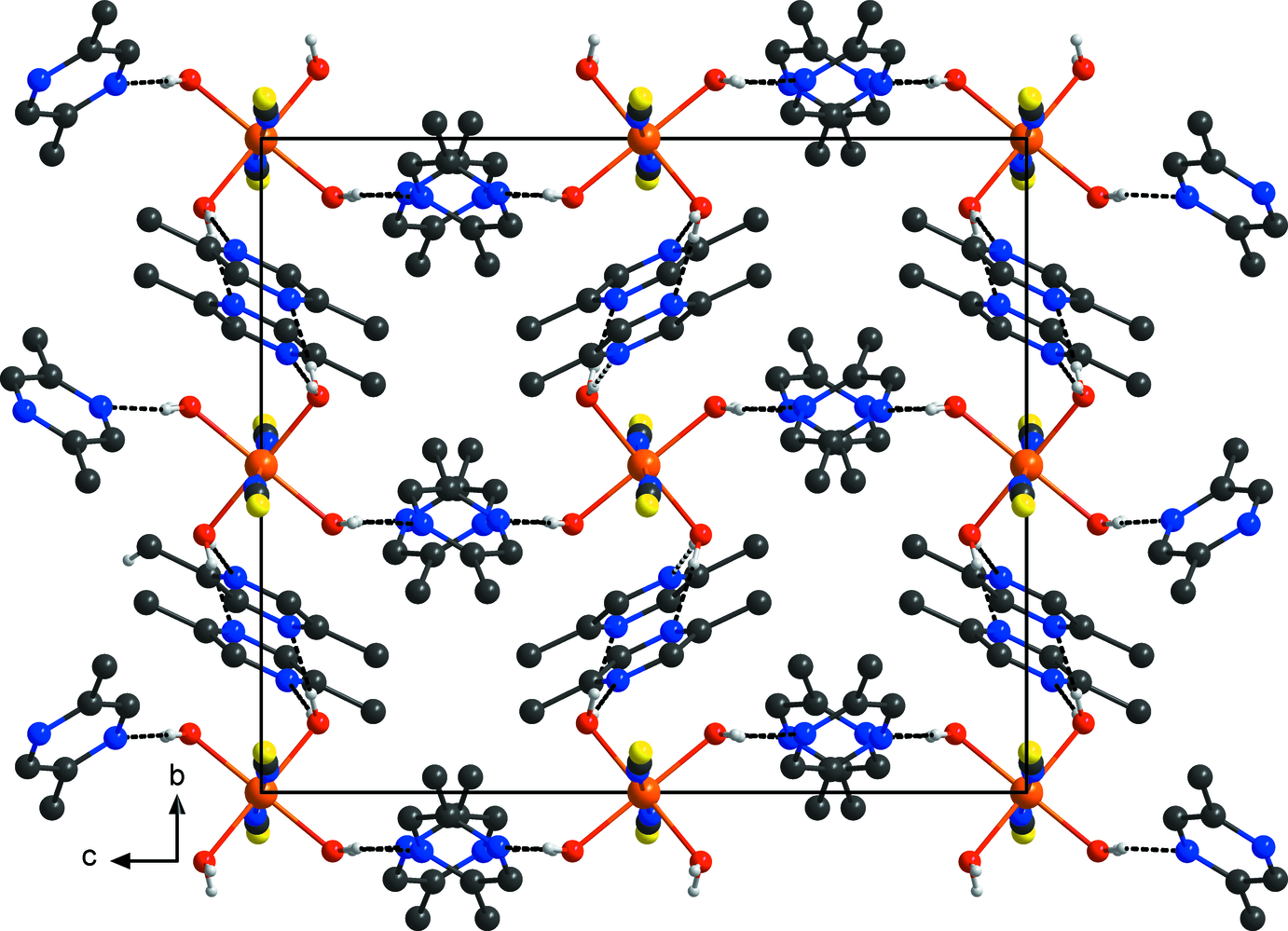

Supplement: Supplementary file 4 [file e-71-00m18-fig2.tif]
